# Gender gaps in research: a systematic review

**DOI:** 10.12688/f1000research.140694.2

**Published:** 2024-01-22

**Authors:** Isabel Cristina Rivera-Lozada, Gissel Carolina Escobar, Oriana Rivera-Lozada

**Affiliations:** 1 Departamento de Economía, Universidad del Cauca, Cauca, Colombia; 2 Facultad de educación, Universidad Nacional Mayor de San Marcos, Lima, Peru

**Keywords:** Gender gaps, discrimination, segregation, research

## Abstract

**Background:**

Despite significant advancements in closing the global gender gap, there is still much progress to be made, particularly in the field of science and scientific research. Numerous studies have addressed this issue and identified a variety of factors that contribute to gender asymmetries in research.

**Methods:**

This study aimed to identify the determinants of gender gaps in scientific research present in the most cited studies of the past ten years as a first step towards closing these differences. Through a systematic literature review that incorporated the Proknow-C Knowledge Development Process and Constructivism methodology.

**Results:**

The results lead us to identify four dimensions to classify the determinants of the gaps in scientific research: academic supply, research policies, scientific production and researcher profile with their respective quantitative or qualitative indicators.

**Conclusions:**

As a potential basis for further modeling that offers greater analytical and correlational depth, as well as the identification of targeted strategies aimed at reducing gender gaps in research.

## Introduction

As indicated by the Global Gender Gap Report 2022, it will take 132 years to close the global gender gap, statistics designed to measure gender equality and inequality such as income, political representation, wealth accumulation, tertiary education levels, stress levels ( World Economic Forum, 2022). In the case of Latin America and the Caribbean, based on the current rate of progress, the region will close the gap in 67 years ( Mujeres 360, 2022). Despite notable advancements in the region, some countries (Argentina, Brazil, and Mexico) appear to have stagnated, while others (Peru, Guyana, and Chile) are improving their gender parity outcomes. At the other end of the spectrum, countries such as Colombia, Honduras, Barbados, and Belize have widened the gender gap. These results contrast with the Sustainable Development Goals, which aim to reduce gender inequalities by 2030 ( United Nations, 2015) and hinder the increase in scientific productivity by 15% to 20% ( International Labor Organization, 2017; Science Metrix Inc., 2018), highlighting the impact that achievements in reducing inequality have on the global economy and closing social gaps.

In recent years, numerous studies have been conducted to investigate differences in scientific productivity ( Abramo *et al*., 2013; López-Bassols *et al*., 2018; Pinho-Gomes *et al*., 2020) in various areas such as psychology ( Mayer & Rathmann, 2018), elite researchers ( Sá *et al*., 2020), science and technology ( Pons *et al*., 2013). Others have focused on identifying achievements and challenges ( Osorio, 2005), obstacles and barriers ( García-Jiménez & Herrero, 2022; Ramírez López & Bermúdez Urbina, 2015; Valenzuela *et al*., 2022), to mention a few. The diversity of research interests exploring aspects of the problem related to the determinants and/or explanatory factors of gender gaps in scientific research leads us to consider it relevant to conduct a comprehensive literature review focused on gender differences in scientific research. The aim is to identify the determinants of gender gaps in scientific research as a first step towards closing these differences.

In order to conduct a relevant literature review, this research focused on a systematic review that incorporated the Proknow-C Knowledge Development Process and Constructivism methodology ( Ensslin *et al*., 2013) to identify potential factors or determinants that make a difference in research for female researchers in the most relevant studies.

This research proposes classifying the determinants into four dimensions: i) Academic Offerings, ii) Research Policies, iii) Scientific Production, and iv) Researcher Profile. The proposed classification allows the recognition of each factor and the definition of indicators, whether quantitative or qualitative, that reflect the situation in the respective field. These indicators serve as a basis for subsequent modelling, offering greater analytical and correlational depth, as well as identifying strategies to address and reduce gender gaps in research.

## Methods

This qualitative research, based on a documentary design, relies on a systematic review and bibliometric analysis, which enables the study of quantitative aspects of production, dissemination, and use of recorded information ( Araújo & Arencibia, 2002; Morales-Morejón & Cruz Paz, 1995). The Proknow-C methodology ( Ensslin *et al*., 2010) is employed, consisting of three stages: Development of the Relevant Bibliographic Portfolio (RBP), bibliometric analysis and systemic analysis. The research proposes to classify the information into 4 factors: i) academic offer, ii) research policy, iii) scientific production and iv) research profile ( Rivera-Lozada *et al.*, 2022).

**I) Development of the Relevant Bibliographic Portfolio (RBP).** The Relevant Bibliographic Portfolio (RBP) refers to the result of the sampling conducted on the relevant scientific literature concerning the gender gap in research. For defining the databases, we established the axes and keywords for the search, as shown in Table 1.

**Table 1.** Definition of Axes and Keywords.

Axes	Keywords
Scientific research	*Research, scientific production, scientific recognition, scientific publications*
Research barriers	*Obstacles or barriers or challenges, scientific research*
Gender gaps	*Gender gap, gender inequality, historical inequality, women*

The databases were selected to enable filtering with Boolean equations in English and/or Spanish, differentiating the type of publication (book or article) and the temporal horizon (2012-2023).

The verification of compliance with these requirements is presented in Table 2.

**Table 2.** Selection of databases.

Databases	Fields	Boolean expressions	Temporal horizon	Type of publication	Last revision date
Scopus	**✓**	**✓**	**✓**	**✓**	2nd week of May
Redalyc	**✓**	**✓**	**✓**	**✓**	2nd week of May
La referencia	**✓**	**✓**	**✓**	**✓**	2nd week of May
Base-search.net	**✓**	**✓**	**✓**	**✓**	2nd week of May
Web of science	**✓**	**✓**	**✓**	**✓**	2nd week of May
Scielo	**✓**	**✓**	**✓**	**✓**	2nd week of May
DOAJ	**✓**	**✓**	**✓**	**✓**	2nd week of May
Ebsco	**✓**	**✓**	**✓**	**✓**	2nd week of May

All documents that provided relevant information on the determinants of scientific research and the gender gaps in it were selected ( Table 2a). The selected documentation was tabulated and classified according to objectives, methodology, variables, population, year of publication, results and conclusions. This classification served as a reference to identify the most pertinent, novel, curious or relevant documents that required special attention.

**Table 2a.** Bank articles according to the database.

	DATA BASE							
Key words	Redalyc		La Referencia		Base-search.net		Scielo	
	Results	Potential results for the study (GBA)	Results	Potential results for the study (GBA)	Results	Potential results for the study (GBA)	Results	Potential results for the study (GBA)
"female researchers "+"barriers "+"gender"	114	29	0	0	6	0	159	1
"research "+"scientific production "+"obstacles" + "women"	56	56	4	0	984	10	3	1
"scientific production" + "inequality"	163	2	0	0	0	0	9	0
"scientific production" + "gender inequality" + "challenges"	6	3	10	0	0	0	0	0
"scientific publications" + "women"	199	199	2	1	1	0	14	14
"profile" +"women researchers"	12	2	0	0	4	1	0	0
"determinants" +"women researchers"	2	1	1	1	1	0	0	0
"perception" +"women researchers"	11	0	10	3	1	1	0	0
"incentives" +"women researchers"	2	2	4	0	4	0	1	1
"scientific research" + "women"	1060	0	4163	0	14	14	67	67
"scientific research" + "women in research"	7	7	8	8	0	0	0	0
TOTAL	1635	292	4202	13	1015	26	253	84

	DATA BASE									
Key words	DOAJ		Scopus Spanish		Scopus English		Web of science		Ebsco	
	Results	Potential results for the study (GBA)	Results	Potential results for the study (GBA)	Results	Potential results for the study (GBA)	Results	Potential results for the study (GBA)	Results	Potential results for the study (GBA)
"female researchers "+"barriers "+"gender"	3	0	0	0	0	0	0	0	90	4
"research "+"scientific production "+"obstacles" + "women"	0	0	3	3	50	50	16	16	0	0
"scientific publications" + "women"	98	98	0	0	3	3	0	0	0	0
"profile" +"women researchers"	1	0	0	0	0	0	0	0	67	3
"perception" +"women researchers"	1	0	0	0	0	0	0	0	65	3
"incentives" +"women researchers"	0	0	0	0	0	0	0	0	60	0
"scientific research" + "women"	871	0	1	1	19	19	8	8	0	0
"scientific research" + "women in research"	6	6	0	0	0	0	7	7	0	0
TOTAL	995	104	4	4	72	72	31	31	282	10

**Table 2b.** Total potential results of each database.

Database	Potential results for the study (GBA)	Total GBA
Redalyc	292	636
La referencia	13	
Base-search.net	26	
Scielo	84	
DOAJ	104	
Scopus	76	
Web of science	31	
Ebsco	10	

Once the mentioned filters were applied, the Gross Bank Articles (GBA) was defined, resulting in 636 articles. The GBA was subjected to an adherence test by calculating a sample with a 95% confidence level and a maximum error of 10% to verify that each article contains at least one of the established keywords ( Table 1).

The formula used is the following:

$$n=\frac{Z^{2}pqN}{{NE}^{2}+Z^{2}pq}$$

Where: *n* is the sample size of the GBA; *Z* is the parameter for the confidence level (1.96 for a confidence level of 95%); *E* is the allowable error (10%); *p* and *q* (50% each); and *N* = 636, being *n* = 83 elements of GBA (13%).

With these conditions, the sample size was determined to be 83 articles to be classified in descending order of citability, along with their main authors, as shown in Table 3.

**Table 3.** Relevant Bibliographic Portfolio (RBP) and respective main authors.

*#*	Author	Citation	*#*	Author	Citation	*#*	Author	Citation
*1*	( Abramo *et al*., 2013)	233	*30*	( Montes & Gallego, 2017)	6	*60*	( Ruiz, 2021)	0
*2*	( Pinho-Gomes *et al*., 2020)	204	*31*	( Torres, 2017)	6	*61*	( González, 2014)	0
*3*	( Mayer & Rathmann, 2018)	100	*32*	( García, 2014)	6	*61*	( Gutiérrez *et al*., 2020)	0
*4*	( Montané & Carvalho, 2012)	54	*33*	( Rojas-Solís *et al*., 2021)	6	*62*	( Barrios & Passerino, 2021)	0
*5*	( Guil Bozal, 2016)	52	*34*	( Caballero Wangüemert, 2016)	5	*61*	( Pérez, 2019)	0
*6*	( Moreno Cubillos *et al*., 2012)	44	*35*	( Sougou *et al*., 2022)	5	*62*	( Contreras *et al*., 2022)	0
*7*	( Stromquist, 2013)	43	*36*	( Ayala, 2019)	5	*63*	( Noguera, 2019)	0
*8*	( Sá *et al*., 2020)	37	*37*	( Guzmán, 2019)	4	*64*	( Figueroa *et al*., 2015)	0
*9*	( Ramos, 2014)	35	*38*	( Vargas *et al*., 2020)	4	*65*	( González, 2015)	0
*10*	( Nygaard & Bahgat, 2018)	34	*39*	( Ferro, 2012)	3	*66*	( Palumbo *et al*., 2022)	0
*11*	( Pons *et al*., 2013)	31	*40*	( Belloso & Sanz, 2017)	3	*67*	( Serrano, 2018)	0
*12*	( Cárdenas Tapia, 2015)	29	*41*	( Al Shammary *et al*., 2021)	2	*68*	( Hernández & Treviño, 2016)	0
*13*	( Centeno-Leguía *et al*., 2020)	15	*42*	( Cruz-Castro, 2021)	1	*69*	( Oliveira *et al*., 2021)	0
*14*	( Calvo & Rodríguez, 2012)	15	*43*	( Florez *et al*., 2016)	1	*70*	( Berggren *et al*., 2022)	0
*15*	( Rodal, 2017)	13	*44*	( Bordons *et al*., 2012)	1	*71*	( Mandıracıoğlu *et al*., 2018)	0
*16*	( Fox *et al*., 2017)	13	*45*	( Burgos, 2013)	1	*72*	( Actis, 2021)	0
*17*	( Davis *et al*., 2022)	13	*46*	( Gomez, 2020)	1	*73*	( Lima *et al*., 2021)	0
*18*	( Zacharia, 2020)	12	*47*	( Lione, 2018)	1	*74*	( Alves, 2013)	0
*19*	( Ramírez López & Bermúdez Urbina, 2015)	11	*48*	( Cervera & Rivera, 2020)	0	*75*	( Franz, 2020)	0
*20*	( Jesús *et al*., 2014)	11	*49*	( Muñoz *et al*., 2021)	0	*76*	( de Oliveira *et al*., 2020)	0
*21*	( Dapía *et al*., 2019)	11	*50*	( Valenzuela *et al*., 2022)	0	*77*	( Del Valle *et al*., 2012)	0
*22*	( Basurto Barcia & Ricaurte, 2016)	11	*51*	( García-Jiménez & Herrero, 2022)	0	*78*	( Quintero & Ibáñez, 2012)	0
*23*	( Vargas *et al*., 2016)	10	*52*	( Castro, 2018)	0	*79*	( García Macas & Ortiz Espinoza, 2014)	0
*24*	( Gnecco, 2016)	10	*54*	( Ochoa, 2017)	0	*80*	( Susinos & Parrilla, 2013)	0
*25*	( Washburn *et al*., 2013)	8	*55*	( Ortiz-Ortega & Sánchez, 2017)	0	*81*	( López-Aguirre & Farías, 2022)	0
*26*	( González & Álvarez, 2016)	7	*56*	( Carrillo Espadas & Flores Galaz, 2023)	0	*82*	( Peñaranda *et al*., 2021)	0
*27*	( Martín, 2012)	6	*57*	( García *et al*., 2019)	0	*83*	( Cirer *et al*., 2020)	0
*28*	( Luna Morales & Luna Morales, 2018)	6	*58*	( Restrepo-Arango, 2016)	0			
*29*	( Truffa, 2012)	6	*59*	( Quintero, 2019)	0			

**Figure 1.**
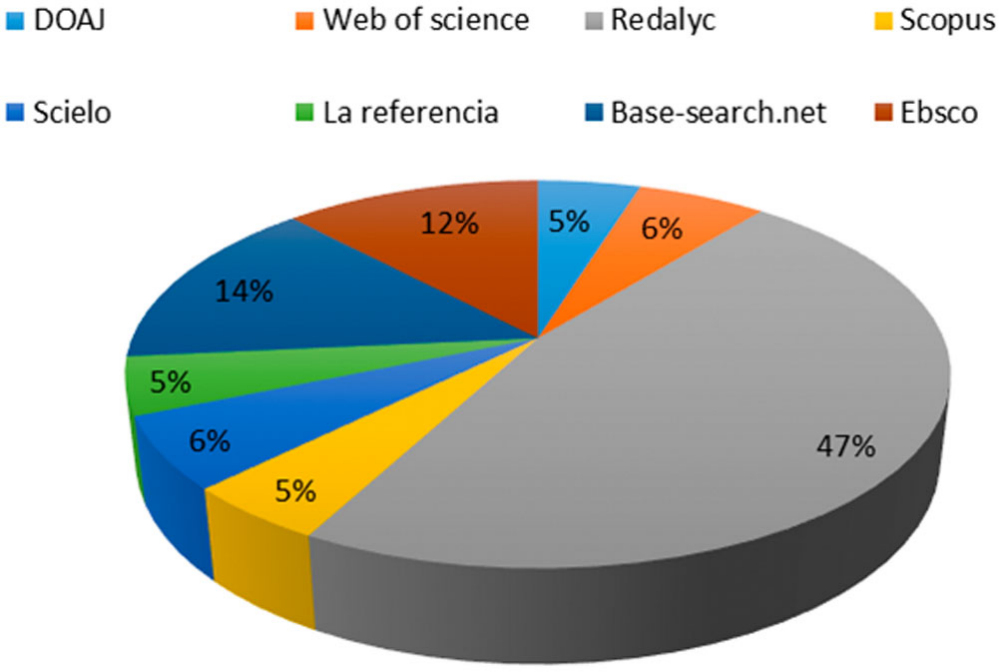
Representativeness test of the RBP.

After the review, it was confirmed that the 83 articles contain the defined keywords in the fields of keywords, title, and/or abstract, verifying adherence. The representation of the used databases in obtaining the RBP is shown in Figure 1. It highlights that the Redalyc database contributes the highest number of articles to the RBP, accounting for 47% of the total. Base-search.net follows with 14%, while the remaining 39% of the RBP is derived from databases such as Web of Science, Scopus, Scielo, La referencia, DOAJ, Ebsco, Redalyc and Base-search.net.

On the other hand, it is possible to evidence representativeness of RBP through citability of the articles. Table 4 shows the list of 21 articles of RBP which stand out due to a citability of 10 or higher. This number is determined to highlight the 25% of RBP.

**Table 4.** Table of highlighted articles.

Year	Authors	Title	Citation
2013	Giovanni Abramo, Ciriaco Andrea D’Angelo, Gianluca Murgiac	Gender differences in research collaboration	233
2020	AnaC. Pinho Gomes, Sanne Peters, *et al*.	Where are the women? Gender inequalities in COVID-19 research authorship	204
2018	Sabrina J. Mayer, Justus M. K. Rathmann	How does research productivity relate to gender? Analyzing gender differences for multiple publication dimensions	100
2012	Montané, Alejandra; Pessoa de Carvalho, Maria Eulina	Diálogo sobre género: justicia, equidad y políticas de igualdad en educación superior (Brasil y España)	54
2016	Ana Guil Bozal	Género y construcción científica del conocimiento	52
2012	Carmen L. Moreno, Luz E. Sepúlveda, Luisa F. Restrepo	Discriminación y violencia de género en la univesidad de Caldas	44
2013	Nelly P. Stromquist	Education Policies for Gender Equity: Probing into State Responses	43
2020	Creso Sa´ ID, Summer CowleyID, Magdalena Martinez, *et al*.	Gender gaps in research productivity and recognition among elite scientists in the U.S., Canada, and South Africa	37
2014	Ana M. González Ramos	¿Camuflaje o transformación? Estrategia profesional de las mujeres en carreras tecnológicas altamente masculinizadas	35
2018	Lynn P. Nygaard a, b, Karim Bahgat	What's in a number? How (and why) measuring research productivity in different ways changes the gender gap	34
2013	Pons Peregort, Olga; Calvet Puig, M. Dolors; Tura Solvas, Marta; Muñoz Illescas, Cristina	Análisis de la Igualdad de Oportunidades de Género en la Ciencia y la Tecnología: Las carreras profesionales de las mujeres científicas y tecnólogas	31
2015	Cárdenas Tapia, Magali	La participación de las mujeres investigadoras en México	29
2020	Dercy Centeno-Leguía a, Luz Morales-Concha b, Crislee E. Lopez c, Christian R. Mejia d	Mujeres científicas: características y factores asociados a la primera autoría y corresponsalía en revistas peruanas indizadas a Scielo, 2010-2015	16
2012	Adelina Calvo Salvador y Carlos Rodríguez-Hoyos	Aportaciones de los estudios de las mujeres y del género a la organización escolar, estado de la cuestión en España.	15
2017	Asunción Bernárdez Rodal	Los estudios universitarios feministas y con perspectiva de género en España (2010-2015)	15
2017	Fox, Mary Franka Send mail to Fox M.F.; Realff, Mary Lynnb *et al*.	International research collaboration among women engineers: frequency and perceived barriers, by regions	13
2022	Pamela B. Davis, Emma A. Meagher, Claire Pomeroy, William L. Lowe Jr, *et al*.	Pandemic-related barriers to the success of women in research: a framework for action	13
2020	Prof. Dr. Zacharias C. Zacharia, Dr. Tasos Hovardas, Dr. Nikoletta X Ms Maria Irakleous	Education and employment of women in science, technology and the digital economy, including AI and its influence on gender equality	12
2015	Dulce Karol Ramírez López, Flor Marina Bermúdez Urbina	Avances, retos y desafíos: aproximación al estado del conocimiento de los estudios de género en educación superior en México	11
2014	María Jesús Matilla Quiza Esperanza Mó Romero	De la excepción a la normalidad: Mujeres científicas en la Historia	11
s.f	María Jesús Matilla Quiza Esperanza Mó Romero	De la excepción a la normalidad: Mujeres científicas en la Historia	11
2019	María Dapía Conde Ricardo Escudero Cid Manuel Vidal López	¿Tiene género la ciencia? Conocimientos y actitudes hacia la Ciencia en niñas y niños de Educación Primaria	11
2016	Johanna Basurto Barcia Carla Ricaurte-Quijano	Mujeres en turismo: Equidad de género en la docencia e investigación en el área de Guayaquil, Ecuador	11
2016	Vargas, Domingo; Requena, Jaime; Caputo, Carlo	Género en la ciencia venezolana: desvanecimiento de la brecha	10
2016	Gnecco-Lizcano, Angela María	Mujeres indígenas: experiencias sobre género e inclusión en la educación superior	10

### Data processing

Regarding the documentation selection process, it took the documents that met all the keywords proposed in the Boolean equation. The review, filtering of this documentation and analysis was done using the Mendeley literature manager Desktop version 1.19.8 and then exported the metadata of the publications obtained from the search were downloaded from the databases in Microsoft Excel format. For the underlying data, see Rivera-Lozada *et al*. (2023a). The phases of definition of the protocol, search and extraction of the initial data from the databases were carried out by all the authors of this publication. The search results are current as of the second week of May. The subsequent filtering of the successive phases was carried out by peer review among the authors.

**Figure 2.**
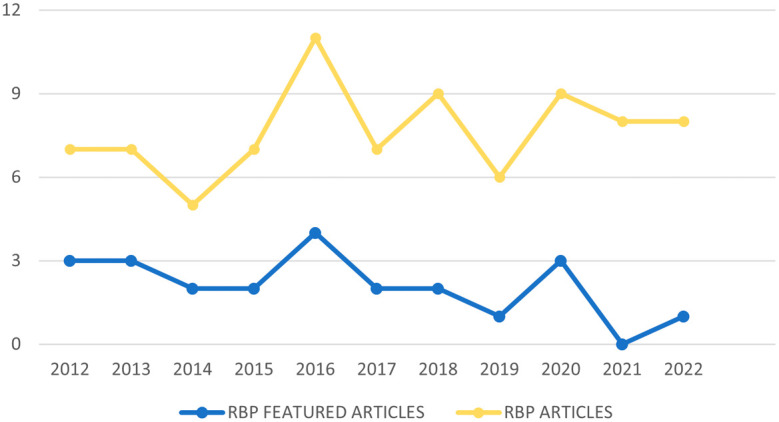
Temporal distribution of RBP articles.

Figure 2 contains the diagram of the process flow carried out to obtain our RBP. In the first instance, duplicate articles and articles that despite carrying out the search determining the interval of years, did not comply with this, are eliminated. Subsequently, those that contain the keywords in the title or in the abstract but any of both shows they are not related to the subject of study are excluded, such as the article “Analysis of the world scientific production on forced sterilization of women with disabilities between 1997 and 2016” ( Concha & Ferrer, 2019), our Keyword: scientific production and women or the article “Women and aging in social research (1950-2018)” ( Torralbo & Guizardi, 2020), our keywords: women and research. Then we obtained reports sought for retrieval but we do not have access to 40 of these, like the “Chapter 4 Gender and Economics in Latin America: a Systematic Analysis of Scientific Production in Scopus” ( Maldonado & Quiñonez, 2021) and finally, the articles are organized from highest to lowest citation ability and the articles that are outside the sample of 83 articles that were determined with the aforementioned formula are excluded.

The systematic review, like other study designs, can present biases that can lead to errors in the study, for which a series of measures were applied to guarantee the objectivity of the results, such as defining a search protocol with quality criteria, as well as searching the two main research databases such as Web of Science, Scopus, Redalyc, among others. Likewise, there were no spatial or language restrictions for the selection of information.

## Results

### Bibliometric analysis

**Figure 3.**
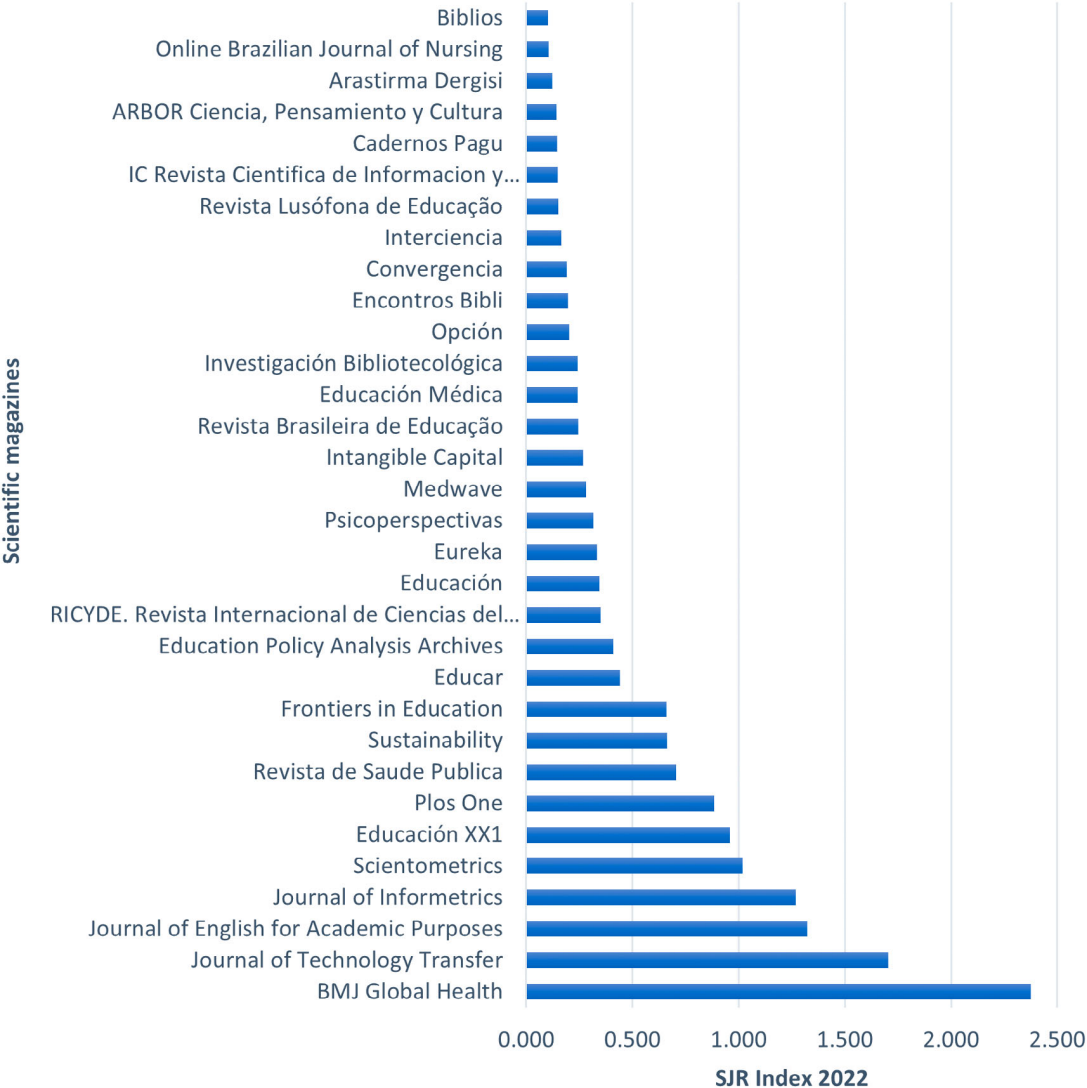
Specialized journals publishing topics related to the gender gap in research.

Figure 3 shows the number of articles from the RBP published in the period 2012-2022, as well as the number of highlighted articles from the RBP based on their citability, published within this time interval. It can be observed that the year 2014 marked a turning point in the decline of publications, reaching its peak in 2016, which also had the highest number of highlighted articles. However, this pattern does not repeat when considering the lowest number of published articles. For the RBP, the year with the lowest number of publications is 2014, while in terms of highlighted articles, the year with the fewest was 2021, where not a single article surpassed 10 citations.

Figure 3 highlights the articles from the RBP according to the journals where they were published. The list includes 32 journals that published the respective articles from the RBP, ordered according to the Scimago Journal Rank (SJR) index, which measures the prestige of the journals based on the citation count received by each publication. Notable journals such as BMJ Global Health, Journal of Technology Transfer, and Journal of English for Academic Purposes stand out with SJR indexes of 2.37, 1.70, and 1.32, respectively. There is a significant difference of 0.67 points between the first and second-ranked journals. However, the subsequent journals show a smaller decrease in their scores, as seen in the difference of 0.38 points between the second and third-ranked journals. The journal with the lowest index is Biblios, with 0.10 points.

Based on these prominent journals, the coverage years of the SJR index for the top 10 journals were identified, as shown in Table 5.

**Table 5.** Coverage years of the SJR index for the highlighted journals in the RBP.

Scientific journal	Years of index coverage
BMJ Global Health	2016-2022
Journal of Technology Transfer	1977-2022
Journal of English for Academic Purposes	2002-2022
Journal of Informetrics	2007-2022
Scientometrics	1978-2022
Educación XX1	2008-2022
Plos One	2006-2022
Sustainability	2009-2022
Frontiers in Education	2016-2022
Educar	2018-2022

Furthermore, the year of the first publication on the gender gap in research was determined for the highlighted journals in the RBP, as shown in Table 6.

**Table 6.** Determination of the year when publications on the gender gap in research began.

Name of the scientific research	First year of publication on the topic
BMJ Global Health	2016
Journal of Technology Transfer	1993
Journal of English for Academic Purposes	2003
Journal of Informetrics	2007
Scientometrics	2002
Educación XX1	2013
Plos One	2012
Sustainability	2014
Frontiers in Education	n.d
Educar	2015

**Figure 4.**
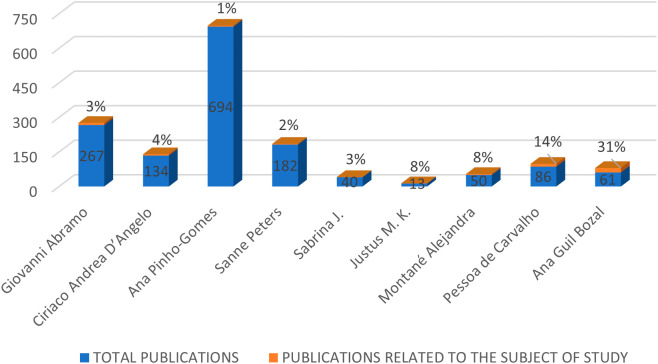
Prominent authors.

Along with the highlighted journals, information about the prominent authors in the RBP was also collected, selecting authors with the highest citability within the RBP, as shown in Figure 4. The figure compares the total number of publications of each author with the publications they have made related to the gender gap in research. Among these authors, Ana Pinho Gomes stands out with the highest number of total publications. Giovanni Abramo, Sanne Peters, and Ciriaco Andrea D’Angelo also have a percentage of their total publications related to the topic of study, with 1%, 3%, and 4% respectively. In contrast, authors such as Pessoa de Carvalho, Ana Guil Bozal, and Alejandra Montané have a smaller number of publications but a higher involvement in the subject at hand, with 14%, 31%, and 8% respectively.

**Figure 5.**
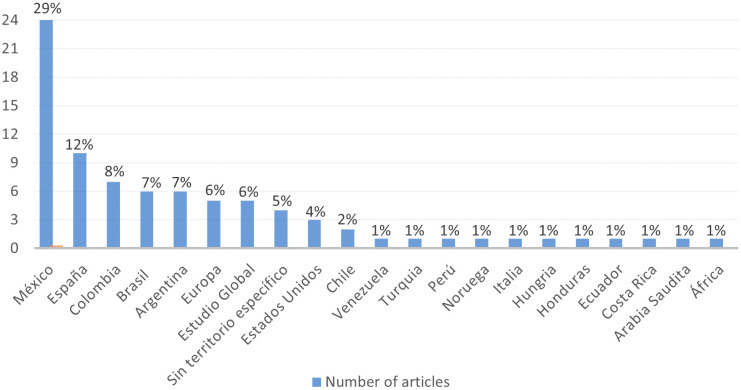
Spatial Focus. Geographical location of studies in the RBP.

It is possible to identify the geographical areas where the studies on the gender gap in research included in the RBP were conducted, as shown in Figure 5.

Based on the information recorded in Figure 5, the evidence indicates that the country with the highest number of studies related to the gender gap in research was Mexico (29%), significantly surpassing other territories. Spanish publications ranked second (12%), followed by Colombian (8%), Argentinean (7%), Brazilian (7%), European (6%), Global Studies (6%), publications without a specific territory (5%), United States (4%), Chile (2%), and to a lesser extent, Venezuela, Turkey, Peru, Norway, Italy, Hungary, Honduras, Ecuador, Costa Rica, Saudi Arabia, and Africa (1% each). This provides the study with global perspectives.

In addition to the spatial focus, the classification of the RBP was incorporated according to the methodological approach of the studies, indicating that 42% were qualitative and 58% were quantitative.

The review made it possible to classify the potential determinants of scientific research: geographical location, position, dedication time, type of call, teaching classification or hierarchy, age, dependency load, inclusive financing funds, sexist bias, gender stereotypes, discrimination, institutional determinants, biases of evaluation committees, income, glass ceiling, scientific recognition or status, salary, motivations, sticky floor, labor contract, marital status, professional segregation, visibility of scientific production, among others.

The systemic analysis of the RBP allowed us to categorize the barriers contributing to the gender gap in research into four dimensions with their respective indicators: i) Academic Offerings: number of higher education institutions total number of careers, female and male enrolment, science careers, female and male enrolment in sciences (natural and social) ii) Research Policies: agency, call for proposals, inclusive funding, evaluation committee, institutional policy, discrimination, recognition or incentive, types of hiring, iii) Scientific Production: research category, publications number, patents, collaboration, segregation, citability, and iv) Researcher Profile: motivations, sticky floor, income, position, geographic location, education level, age, gender, marital status, dependency load, scale, acknowledgment.

**i) Academic offerings:** Ten percent of the RBP focused on explaining or analyzing gender gaps in research based on considerations that incorporate variables such as the number of educational institutions, professional careers offered, total enrolment, STEM (Science, Technology, Engineering, and Mathematics) careers, and enrolment of women in natural sciences or STEM. In this regard, the results presented by López-Bassols *et al*. (2018) indicate that beyond acknowledging the existence of gender gaps in research, it is necessary to identify the areas or fields of knowledge where these gaps are most significant in order to correlate them with women’s participation and presence in higher education across various disciplines. Research conducted for Latin America and the Caribbean identifies family pressures, stereotypes, expectations, lack of mentors and role models, as well as vertical segregation and the “leaky pipeline” phenomenon as causal factors contributing to the existing differentials in women’s participation in research.

The authors propose considering three dimensions: actors within the national science and innovation system (higher education institutions, government, companies, NGOs), activities related to science and technology (teaching, research, publications, patents, funding, and innovative entrepreneurship), and obstacles or motivations (opportunities, attitudes, financial support, other incentives, role models, discrimination, and social biases). Based on the aforementioned, they establish a set of 16 indicators classified under higher education, careers in science and technology, scientific research, and innovation and innovative entrepreneurship.

On the other hand, the research conducted by García and Ortíz (2014) explores the individual and institutional determinants affecting gender gaps in scientific production in Ecuadorian universities. Using a linear regression model, they propose individual determinants that include academic degree, number of scholarship recipients, hours of dedication, age, and gender, as well as institutional determinants that incorporate the number of R&D projects, laboratories, executed budget for R&D, number of collaborators, among others. The research results indicate that institutional determinants have a significant and positive impact, while the number of PhDs still does not have a significant effect on scientific production. In particular, they find that women are less productive than men and that the age range with the highest productivity is between 30 and 39 years. Additionally, they found that the number of collaborators has a negative effect.

The UNESCO study on STEM education (2019) identifies four factors affecting gender gaps in research based on women’s participation in STEM education: individual factors (biological and psychological), family factors (parents’ beliefs, parents’ level of education, household socioeconomic status, and other characteristics), school factors (teaching staff, pedagogical strategies, teachers’ perceptions, interactions with students, textbooks, educational materials, curriculum, STEM equipment and resources, evaluation strategies and tools), and social factors (gender equality, social norms, policies and legislation, media and social communication).

The document debunks the belief in biological differences in the brains of men and women as an explanatory factor for their participation in STEM disciplines and research in this field. In this regard, neurological plasticity, understood as the brain’s ability to create new connections, is the essence of the learning process. The self-selection bias is the reason why girls and women decline STEM education, and this selection is influenced by stereotypes and androcentric biases acquired during upbringing and socialization moments. Similarly, social norms and stereotypes disseminated by the media have a significant impact on the internalization of gender roles, occupations, skills, and capabilities by girls and boys ( UNESCO, 2019).

Based on the literature review conducted, the indicators in the dimension of Academic Offerings are:. Based on our review of the literature, we identified the following indicators: number of higher education institutions, total number of offered careers, number of careers in science, total enrolment, total female enrolment, total male enrolment, enrolment of women and men in Natural Sciences, enrolment of male and female in Social Sciences, and enrolment of women and men in Health Sciences ( Carrillo & Florez, 2023; Del Valle *et al*., 2012; Ortiz-Ortega & Sánchez, 2017).

**ii) Research policies:** Twenty-nine percent of the RBP disseminated results regarding the impact of research policies on gender gaps in research. Mexico is the country where the highest number of studies in this field has been conducted, and thus, the equity of gender in research was evaluated in 2012, 2013, and 2015 ( Cárdenas Tapia, 2015). With a database that provided gender-specific information, knowledge area, SNI (National System of Researchers) classification, and public universities, it was possible to demonstrate that the participation of female researchers in Mexico is lower than that of men, and they also have a lower level in the SNI in all categories. In more detail, women are not the majority in any of the seven knowledge areas proposed by the SNI. Despite this, from highest to lowest, women are found in 1) biology and chemistry, 2) humanities and behavioural sciences, 3) social and economic sciences, 4) physics, mathematics, and earth sciences, and 5) engineering.

The results confirm the scissor effect or pyramid effect regarding women’s participation in science, as the number of women decreases as their professional career progresses. This situation demands effective policies that encourage and ensure greater presence and participation of women in scientific research in Mexico.

On the other hand, the research by González and Álvarez (2016) aimed to determine the factors that influence the achievement of efficient research formulation in Mexico through a descriptive-correlational study. With a sample of 42 researchers, they identified the following factors influencing the achievement of research formulation: development of analytical thinking, efficient database searching, management of research projects, efficient use of software, data analysis and modelling, innovation, and proper time management.

Pons-Peregort *et al.* (2013) analyzed gender equality of opportunities in science and technology to understand the career paths of female scientists in Spain. Using a mixed methodology, they identified that equality of opportunities in internal promotion, salary disparities, and work-life balance are challenges that can be overcome with research policies. The low presence of women in scientific professional careers is based on the hegemony of masculine values, which calls for rethinking the achievement of the critical mass needed to bring about structural transformations in the research field, estimated to be 35% ( Langford *et al*., 1995). The authors propose policies that support maternity, childcare services, tax deductions for women who stay at home to care for children because their absence perpetuates women’s inferior position in the labor market and keeps them away from the research field.

In this sense, the indicators that contribute to the definition of the dimension of research policies are entities proposing the policy (State, Ministries, Universities, NGOs), research calls, inclusive financing, composition of the evaluation committee, institutional university policy, incentives and/or recognition, organizational culture (discriminatory biases), types of contracts.

**iii) Scientific production:** Twenty-four percent of the RPB aims to study the gender gap in scientific production. Luna & Luna (2018) analyzed Mexico’s scientific production recorded in Web of Science from 1900 to 2000 in the fields of exact sciences and engineering to characterize the involvement of female researchers in these fields of study. As these areas have traditionally been associated with men, the research sought to highlight the breakthroughs and achievements of women, considering that they are not the majority in either field. The research used indicators in regard to gender, scientific production and impact, bibliometric and co-authorship network analysis.

The results showed that scientific production increased from the 1980s and 1990s, mainly due to increases in postgraduate studies, the consolidation of research groups, and an increase in national and international scientific collaborations ( Luna & Collazo, 2002). Scientific production is concentrated in five institutions and is led by UNAM, which has had female representation for 29 years. Additionally, the co-authorship network of research groups in the physical, chemical, mathematical, and engineering studies emerges in various specialties. The increase in scientific productivity is associated with the growth of female enrolment in higher education in natural sciences (47%) and engineering (25%) ( UNESCO, 2019), as well as the creation of new educational institutions since the 1960s and the emergence of women in traditionally male-dominated disciplines.

Continuing with Mexican research, Castro (2018) explores how a group of female researchers breaks paradigms and reshapes the line of women in scientific production. To achieve this, they incorporated qualitative-quantitative methods that included Participatory Action Research (PAR), Véster’s sensitive model, as well as validation indexes and indicators; monitoring and prospecting systems. This allowed them to conclude that women construct knowledge supported by a real and symbolic world.

García (2014) explores the situation of women in the research field in Mexico and discovers that difficulties arise in reconciling academic degrees with motherhood, publishing requires fulfilling the double workload, supervising degree projects requires time and mobility availability, lack of transparency in selection processes, and being evaluated by men negatively impact women’s research.

In Ecuador, Basurto and Ricaurte (2016) used a mixed study to examine the situation of women in the academic field of tourism. The results identified the low representation of women in teaching positions (53%) compared to the percentage of female students in tourism (75%), as well as male leadership in research from participating educational institutions, which are mostly organized by women. The explanatory factors found were difficulty reconciling research roles with motherhood, gender stereotypes, and the social perception of tourism as a feminine disciplinary field.

López and Farías (2022) propose a quantitative analysis of the temporal trajectories of gender parity in scientific publications in Colombia. The country ranks fifth in scientific productivity in Latin America and allocates 0.5% of its GDP to R+D ( MINCIENCIAS, 2020). English language proficiency is a disadvantage for Colombian researchers and is closely related to socioeconomic status. Similarly, gender stereotypes in the workplace negatively affect female researchers, leading the authors to assert that it is not simply about increasing the number of women in science and their scientific production, but rather reevaluating how science is done and valued in Colombia, which requires an inclusive and equitable ecosystem.

They highlight that the scientific areas with the highest number of female publications are medical sciences (37.76%), social sciences (35.51%), and natural sciences (29.09%).

Regarding gender gaps in research productivity and recognition among elite scientists in the United States, Canada, and South Africa, as studied by Sá *et al*. (2020), it was found that women in science in these countries are under-cited, underpaid, underpromoted, and receive less professional recognition compared to their male counterparts, which puts women at a disadvantage when considering the principle of cumulative advantage, indicating that greater recognition leads to more productivity. They identified factors such as differences in family responsibilities, different patterns in the use of time (women spend more time teaching, advising students, and participating in committees), unequal allocation of resources, gender bias in peer review, gender stratification in disciplines, as well as different patterns in academic collaboration and network building as explanatory factors for the low productivity of female researchers.

In an Italian study conducted by Abramo *et al*. (2013) using a bibliometric approach, they sought to identify academics’ propensity for collaboration, leading them to conclude that women demonstrate a greater capacity to collaborate in all the analysed forms (intramural, extramural, national, and international), except in international collaboration, where the gap with male counterparts persists.

Among the explanatory factors for productivity gaps in research compared to men, they found that the low percentage of female academics, discrimination affecting job opportunities, biases, difficulty accessing funding, and limitations due to family responsibilities negatively affect female researchers.

Pinho-Gomes *et al*. (2020) investigate the authorship of women in COVID-19 research by asking “where are the women?” The study shows that women are underrepresented in research, particularly in first and last author positions. These gender biases point to broader inequalities that include authorship in other scientific areas and senior authorship.

Based on the aforementioned, the indicators in the scientific production factor are the following researcher category, number of publications, number of patents, collaborative authorship, type of publication (scientific article, popular article, book chapter, book, collections), segregation (female collaborations), and citability.

**iv) Profile.** Thirty-seven percent of the RBP presented research results addressing the characteristics, motivations, uniqueness, and distinctive traits of researchers. In this perspective, Prieto de Alizo (2008) provides a theoretical approach to the characteristics of researchers in the humanities field using a phenomenological, hermeneutic, and ethnographic approach with successive interviews until theoretical saturation of categories is reached. The study conducted in Venezuela defined researchers as individuals with an active interest in understanding, learning, deconstructing, and constructing their reality. Beyond defining the characteristics of the group of researchers, the research made it possible to address two other fundamental aspects: the researchers’ conception of conducting research and the context in which research is carried out. This includes aspects such as institutional identification, the academic space where they work, the research-teaching relationship, the appreciation of the profession, administrative strategies in terms of institutional and state policies, as well as quality criteria.

In Spain, Dapía *et al*. (2019) explored whether science has a gender association among primary school students. Using a gender perspective analysis, they administered the PANA instrument (Project on Attitudes Toward Science in Children and Adolescents) by Pérez and Pro (2005) and the ROSE questionnaire ( Schreiner and Sjøberg, 2004) to 378 students. The investigation showed a weak association between gender and science, a more positive attitude towards science among boys, and no gender bias in the desire to become a scientist. This led to the conclusion that there is a need to improve knowledge about the contributions of science and that career aspirations to become scientists in primary education are not associated with gender.

Research conducted in Mexico by Carrillo and Flores (2023), aimed at describing the educational trajectory of women during their professional studies, surveyed 152 women scientists from the National System of Researchers (SNI) selected through non-probability sampling. The study aimed to identify obstacles, challenges, and experiences in the scientific field. Using descriptive quantitative methodology, they found that women face gender-related barriers such as invisibility, lack of recognition for scientific contributions, inequity, stereotypes associated with the care economy, dual burden of work, and difficulties in achieving work-life balance.

In the same vein, they identified variables associated with gender such as the choice of professional career, gender division of labour, sociocultural conditions, social valuation, stereotypes, and gender roles. Regarding the academic trajectory, the variables that influence it are discipline, postgraduate studies, periodic publications, difficulties in entering and advancing in the SNI (lower hierarchy, prolonged stagnation, institutional conditions, as well as symbolic and social gender aspects).

As a result, the main challenges faced by female researchers are related to bureaucracy, in terms of paperwork and procedures, the evaluation system based on quantity rather than quality, funding, lack of job security, competition in what they call the “wolf environment,” excessive workload due to lack of staff, and career advancement understood as continuous training and development processes.

Therefore, the selected indicators for the profile dimension are motivations, existence of a sticky floor, income level, position, geographic location, educational level, career, age, gender, marital status, dependent care burden, rank or category, and recognition.

## Conclusion

Research focused on gender differences in scientific research has identified a diverse range of determinants or explanatory factors for these gaps. Through a systematic review using the Proknow-C Knowledge Development Process and Constructivism methodology, this research identified the most relevant studies and potential factors influencing scientific research for female researchers and academics.

The research facilitated the identification of relevant indicators grouped into four dimensions. The first dimension analysed was academic offerings, considering variables such as the number of educational institutions, offered professional careers, total enrolment, STEM careers, and enrolment of women in natural sciences or STEM. Additional indicators included total female enrolment, female enrolment in natural sciences, female enrolment in social sciences, and female enrolment in health sciences.

The second dimension examined was research policies. The research found that 29% of the Relevant Bibliographic Portfolio (RBP) presented results regarding the impact of research policies on gender gaps in research. Mexico stood out as the country with the highest number of studies in this field. Indicators considered in the analysed documents included policy-proposing organizations (government, ministries, universities, NGOs), research calls, inclusive funding, composition of evaluation committees, university institutional policies, incentives/recognition, organizational culture (discriminatory biases), and types of employment contracts.

The third dimension focused on scientific production. The research found that 24% of the RBP aimed to study the gender gap in research production. The selected indicators were researcher category, number of publications, number of patents, collaborative authorship, publication types (scientific articles, popular articles, book chapters, books, collections), gender segregation in collaborations, and citability.

The final dimension addressed the profile of researchers. Approximately 37% of the RBP included studies investigating characteristics, motivations, singularities, and distinctive traits of researchers. Qualitative and quantitative indicators were selected, including motivations, the existence of glass ceiling, income level, position, geographical location, educational level, field of study, age, gender, marital status, dependency burden, rank or category, and recognition.

Systematization processes in the literature review are usually processes whose complexity becomes more evident in the discussion of the results. In particular, because in this case the largest number of documents was concentrated in a single country, Mexico, a situation that leads to conceptual and, above all, contextual biases.

The proposed dimensions of analysis, along with the derived indicators from the conducted research, aim to contribute to the development of explanatory models for the determinants of gender differentials in research. Furthermore, this research aims to help the formulation of effective public policies that address and reduce these gender gaps.

## Data Availability

Zenodo: Gender gaps in research: Literature review, https://doi.org/10.5281/zenodo.8206068 ( Rivera-Lozada *et al*., 2023a). This project contains the following underling data:
• METHODOLOGY.xlsx Zenodo: PRISMA checklist and flow diagram for ‘Gender gaps in research: a systematic review’, https://doi.org/10.5281/zenodo.8267629 ( Rivera-Lozada *et al*., 2023b). Data are available under the terms of the Creative Commons Attribution 4.0 International Public License (CC-BY 4.0 International).
