# Supplementary material for: Gender gaps in research: a systematic review
Source: F1000Res. 2024 Feb 19;12:1302. Originally published 2023 Oct 11. [Version 3] doi: 10.12688/f1000research.140694.3 (PMC11208858; doi:10.12688/f1000research.140694.3)
Supplement: Peer review history

# Reviewer report: Reviewer response for version 3

**DOI:** 10.5256/f1000research.162708.r215196

**Published:** 2024-06-26

**Authors:** Salomon Huancahuire-Vega

**Affiliations:** 1 Peruvian Union University UPeU, Lima, Peru

This is a very interesting work that aims, through a systematic bibliographic analysis, to identify the determinants of the gender gap in scientific research. The work is well structured, written, and presented. I only have a few comments:

 1. There is an apparent database selection bias (despite having included Scopus and WoS, there is an inclination towards regional databases that publish in Spanish and Portuguese, but databases with a different language have not been considered to English). Because of this, I suggest that this limitation of the study be mentioned.

 2. Be careful with the range of years of the bibliographic analysis. In the methodology section, it says 2012 - 2023 but in the results they mention 2012 - 2022

 3. Could you mention the exact Boolean equation? Table 1 has keywords but the equation is not shown.

 4. I do not understand why to calculate the sample size if the complementary materials show that they have followed the Prisma methodology.

 5. In the Methodology - data processing section, it says Figure 2 contains the diagram of the process flow carried out to obtain our RBP. However, Figure 2 doesn't look like a flowchart, why not use the Prism flowchart instead?

 5. Correct in the results section the first paragraph says figure 3 but it seems to refer to figure 2.

 6. In my opinion, tables 5 and 6 and figure 4 are not of major relevance to what this study proposes. The journal, its SJR, the researcher, do not have greater relevance in the identification of determinants of gender gap in research.

 7. On page 12 it says: The systemic analysis of the RBP allowed us to categorize the barriers contributing to the gender gap in research into four dimensions with their respective indicators... I consider this to be the main result of the study, on which you should mention more details of how this determination was reached. All the previous results are analyzes of bibliometric aspects. The latter comes from a review of the content of the selected works, from which these 4 dimensions are determined.

 8. There is no mention of a discussion section. I think the discussion has been going on since he begins to describe the 4 dimensions on page 12.

 9. It would be good to mention the limitations of the study

Are the rationale for, and objectives of, the Systematic Review clearly stated?

Yes

Is the statistical analysis and its interpretation appropriate?

Not applicable

If this is a Living Systematic Review, is the ‘living’ method appropriate and is the search schedule clearly defined and justified? (‘Living Systematic Review’ or a variation of this term should be included in the title.)

Yes

Are sufficient details of the methods and analysis provided to allow replication by others?

Yes

Are the conclusions drawn adequately supported by the results presented in the review?

Yes

Reviewer Expertise:

public health

I confirm that I have read this submission and believe that I have an appropriate level of expertise to confirm that it is of an acceptable scientific standard, however I have significant reservations, as outlined above.

# Reviewer report: Reviewer response for version 3

**DOI:** 10.5256/f1000research.162708.r248381

**Published:** 2024-05-03

**Authors:** Yolvi Javier Ocaña Fernandez

**Affiliations:** 1 UNIVERSIDAD NACIONAL MAYOR DE SAN MARCOS, Lima, Peru

I have read this latest version very carefully, and as I have commented since the first review, the article is very well structured from a methodological point of view, complying with all the characteristics that a systematic review article should have, the topic is very interesting and relevant. in the line of gender research, which is why I once again congratulate the authors for such an excellent work.

Are the rationale for, and objectives of, the Systematic Review clearly stated?

Yes

Is the statistical analysis and its interpretation appropriate?

Yes

Are sufficient details of the methods and analysis provided to allow replication by others?

Yes

Are the conclusions drawn adequately supported by the results presented in the review?

Yes

Reviewer Expertise:

Education and invation

I confirm that I have read this submission and believe that I have an appropriate level of expertise to confirm that it is of an acceptable scientific standard.

# Reviewer report: Reviewer response for version 3

**DOI:** 10.5256/f1000research.162708.r248382

**Published:** 2024-04-26

**Authors:** Lorenzo Escot

**Affiliations:** 1 Complutense University of Madrid, Madrid, Spain

I have reviewed the three versions of the article with interest, care and dedicating the time necessary for its rigorous revision. But the authors' response to the problem of sample selection is clearly insufficient. They clearly recognized in their response that there is a Latin American bias to their systematic review, but they have not revised the article in line with this problem. The authors stated in their comments that this selection bias “was added in the limitations of the article, since incorporating more sample would involve changing results, discussion, conclusions, among others”.

 They also recognize that “the results cannot be generalized to all countries although it makes a good reading of what happens in Latin America and definitely cannot be generalized to all times that are so changing and respond to situational contexts”.

 The problem is that they recognize that all their results are biased in the referee response but not in the main text of the new version 3 of the paper.

 A systematic review of Latin American literature is a very valuable exercise when it is made explicit that it is an analysis of Latin American literature, and it is not intended to be a systematic analysis of "all" literature and generalized to all countries. That is all.

 The paper should make this selection bias problem explicit from the beginning, in the title, in the abstract, in the introduction, in the methodology, in the results - in fact I have not seen anywhere in the paper that they have made explicit reference to this problem. Only at the end of the conclusions is there a brief, insufficient, and not at all clear and explicit, reference to this problem (Is the inclusion of this sentence at the end of the conclusions the incorporation of the limitations referred to by the authors in their response?). This is not acceptable for an article that claims to be systematic, serious, rigorous and scientific.

 In fact, the entire introduction and description of the methodology should be changed to make it explicit, and not hide the existence of this strong bias until the last sentence of the conclusions. This has been the response of the authors to my comments to version 2, to insert at the end of the conclusions a short, vague, and imprecise sentence about the problem of sample selection bias in their entire analysis. The problem of sample selection bias is not a conclusion to your work, it is a limitation of your entire analysis that should be acknowledged and made explicit from the outset.

 In this sense, the title of the paper "Gender gaps in research: a systematic review" is misleading and should be changed to one that reflects the bias "Gender gaps in research: a Latin American literature systematic review", or other that explicitly recognizes the bias in the selection of the literature.

 Also, it has to be explicit in the description of methodology (Methods, Data processing) and in Bibliometric analysis results, not only in the final sentence of the conclusion. Because otherwise, as it now appears at the end of the conclusions, it seems that it is something that authors do not want to recognize. As I have just said, a review of Latin American literature is a very valuable exercise when it is made explicit that it is an analysis of Latin American literature, and it is not intended to be a general and complete systematic analysis of "all" literature.

 After these changes that clear acknowledge at the outset and throughout the title, the methodology and data description, the existence of this bias, then the article will be ready to be accepted for indexing.

 So I have to maintain my decision of 'Approved with reservations', and I insist, because of the insufficient authors' response to solve the problem (or make explicit) the recognized problem of selection bias in their research.

 This is just my opinion. And I am sorry for any inconvenience my opinion may cause to the authors. I understand the need for all of us in academia to publish in scientific journals. But I think it is my duty to try to be serious and rigorous when one accepts the review of scientific papers. Article review processes should be a dialogue and an exchange of ideas and opinions, in which the arbitration of an editorial team is always positive, or at least that is how I understand it. And in this sense, I am open to further discussion with the authors on the need or not to address the issue of sample selection in their article.

Are the rationale for, and objectives of, the Systematic Review clearly stated?

Yes

Is the statistical analysis and its interpretation appropriate?

Yes

Are sufficient details of the methods and analysis provided to allow replication by others?

Yes

Are the conclusions drawn adequately supported by the results presented in the review?

Yes

Reviewer Expertise:

Applied Economics, Gender Economics

I confirm that I have read this submission and believe that I have an appropriate level of expertise to confirm that it is of an acceptable scientific standard, however I have significant reservations, as outlined above.

# Reviewer report: Reviewer response for version 2

**DOI:** 10.5256/f1000research.161374.r239456

**Published:** 2024-02-12

**Authors:** Lorenzo Escot

**Affiliations:** 1 Complutense University of Madrid, Madrid, Spain

I have been able to read the authors' response to my comments. I don't think they really answer my questions. 

 For example, they have added table 2a with the queries, but they do not show the results from the main database they claim to use (Scopus). Maybe I am wrong, but I have not been able to identify this database in table 2a. Perhaps you could clarify this (I have run a Scopus query with "scientific publications" + "women" and got 502 potential papers). Authors states that "These repositories were filtered with the keywords shown in table 2a and it is precisely there where Redalyc presents the largest number of documents related to the keywords of interest for this research". I do not see Scopus in table 2a, I am sure I am missing something because I do not see any result both in Scopus or WoS in this Table2a.

 Also, authors states:  "Perhaps another type of Boolean expressions in search offers repository participations different from those presented in this document. According to the above, we consider that there is no selection bias."

 I think this statement is not accurate. In my opinion, the paper has a large sample selection bias precisely because of the type of queries selected and the type of databases selected. Then the systematic review is not representative of all the literature about gender gaps because its selection bias.

 In any case, the authors do not answer my question "Do the authors think that their results could be generalized to all countries and to any time? Would the resulting papers selected change if other repositories had been used?"

 This bias is important because the analysis of the results has mainly analysed Spanish-speaking authors, is this correct? are they the ones with the most citations, and what about English-speaking authors?

 In any case, I think it is not the time to redo the analysis now. So, I maintain my congratulations to the authors and also my decision "approved with reservations".

Are the rationale for, and objectives of, the Systematic Review clearly stated?

Yes

Is the statistical analysis and its interpretation appropriate?

Yes

Are sufficient details of the methods and analysis provided to allow replication by others?

Yes

Are the conclusions drawn adequately supported by the results presented in the review?

Yes

Reviewer Expertise:

Applied Economics, Gender Economics

I confirm that I have read this submission and believe that I have an appropriate level of expertise to confirm that it is of an acceptable scientific standard, however I have significant reservations, as outlined above.

# Response

**Published:** 2024-02-13

**Authors:** ORIANA RIVERA LOZADA

**Affiliations:** Universidad Nacional Mayor de San Marcos, Lima District, Lima Region, Peru

Dear Reviewer

 We apologize for not having answered the questions appropriately. Surely the selection of Boolean expressions and repositories privileged Spanish-speaking authors in consideration of the subjectivities that are implicit in the perception and situated knowledge that we as Latin American researchers may have. In this case we share the assertion that searches in Scopus and WOS repositories with Boolean expressions in English will yield a greater number of literature, which would lead to new results that demand other analyses.  Therefore, this was added in the limitations of the article, since incorporating more sample would involve changing results, discussion, conclusions, among others.

 In spite of the above, we believe that the results cannot be generalized to all countries although it makes a good reading of what happens in Latin America and definitely cannot be generalized to all times that are so changing and respond to situational contexts.

# Reviewer report: Reviewer response for version 1

**DOI:** 10.5256/f1000research.154078.r215190

**Published:** 2023-12-18

**Authors:** Yolvi Javier Ocaña Fernandez

**Affiliations:** 1 UNIVERSIDAD NACIONAL MAYOR DE SAN MARCOS, Lima, Peru

The research addresses a very interesting and relevant topic at this time as is the identification of the determinants of gender asymmetries in scientific research and provide ideas on how to close these gaps.

 The dimensions they used to classify gender gaps: academic supply, research policies, scientific production and researcher profile, seem to me appropriate and based on the existing literature, so I congratulate the authors for their choice.  The tables and figures help to understand all the information in the article. The conclusions are clear, well-structured and in accordance with the research objectives. The bibliography is well selected and well used throughout the document.

 Additionally, the document is well structured and well written. As a suggestion I propose:

 1. Consider the inclusion of more articles from other databases, in order to avoid some biases, such as selection bias. It could also help to broaden the discussion of the research.

 2.       Expand the introduction so that references from other countries can be included, so that this valuable information can be used in other countries and especially background information related to the dimension of the research.

 Finally, I would like once again to congratulate the authors for their excellent work.

Are the rationale for, and objectives of, the Systematic Review clearly stated?

Yes

Is the statistical analysis and its interpretation appropriate?

Yes

Are sufficient details of the methods and analysis provided to allow replication by others?

Yes

Are the conclusions drawn adequately supported by the results presented in the review?

Yes

Reviewer Expertise:

Education and invation

I confirm that I have read this submission and believe that I have an appropriate level of expertise to confirm that it is of an acceptable scientific standard.

# Response

**Published:** 2024-01-17

**Authors:** ORIANA RIVERA LOZADA

**Affiliations:** Universidad Nacional Mayor de San Marcos, Lima District, Lima Region, Peru

1. Consider the inclusion of more articles from other databases, in order to avoid some biases, such as selection bias. It could also help to broaden the discussion of the research.

The repositories used for the research were, in order: Scopus, Redalyc, La referencia, Base-Search.net, Web of Science, Scielo, DOAJ and Ebsco, considering that they met the criteria of fields Boolean expressions, temporal horizon and type of publication (Table 2). These repositories were filtered with the keywords shown in table 2a and it is precisely there where Redalyc presents the largest number of documents related to the keywords of interest for this research.

 Perhaps another type of Boolean expressions in search offers repository participations different from those presented in this document. According to the above, we consider that there is no selection bias.

 2.       Expand the introduction so that references from other countries can be included, so that this valuable information can be used in other countries and especially background information related to the dimension of the research.

 are included in the text

# Reviewer report: Reviewer response for version 1

**DOI:** 10.5256/f1000research.154078.r217668

**Published:** 2023-11-22

**Authors:** Lorenzo Escot

**Affiliations:** 1 Complutense University of Madrid, Madrid, Spain

The paper provides a systematic review that aims to identify the determinants of gender asymmetries in scientific research and provide insights on how to close these differences.

 The subject is of great interest and relevance. The study identified four dimensions to classify the determinants of gender gaps in scientific research: academic supply, research policies, scientific production, and researcher profile.

 The document appears to be well structured and provides detailed information on how the systematic review has performed. 

 1) My main drawback with respect to the systematic analysis of the literature is in the selected databases. There is a clear sample selection bias towards Latin American and Hispanic journals as a consequence of the inclusion of the Redalyc index, since almost 50% of the bibliographic references found come from this repository. 

 Do the authors think that their results could be generalized to all countries and to any time?. Would the resulting papers selected change if other repositories had been used?

 2) In some cases, the information on the characteristics of the sample of bibliographic references obtained does not allow relevant conclusions to be drawn. For example, Table 5 shows the Coverage years of the SJR index for the highlighted journals in the RBP. I understand that it is total coverage years in SJR, not the years used in the RBP. Is the information in this table really relevant? this information is only of the 5% of the RBP used?, are all the journals and all the papers included in SJR?

 3) Section "iii) Scientific production" summarizes the main findings of a selection of articles. It is not clear what criteria were used to select the articles explained in this section. How were they selected?, are they the most cited articles? are those published in the highest impact journals? or is it a purely random selection?

 It is not clear, therefore, how the factors explained in section iii) are select as the main factors behind the gender gap. Are these factor in scientific literature  supported by the quality of the journal?, or by the number of cites?, or whether this is simply a purely historical review?.

 In any case, I would like to congratulate the authors for their excellent work.

Are the rationale for, and objectives of, the Systematic Review clearly stated?

Yes

Is the statistical analysis and its interpretation appropriate?

Yes

Are sufficient details of the methods and analysis provided to allow replication by others?

Yes

Are the conclusions drawn adequately supported by the results presented in the review?

Yes

Reviewer Expertise:

Applied Economics, Gender Economics

I confirm that I have read this submission and believe that I have an appropriate level of expertise to confirm that it is of an acceptable scientific standard, however I have significant reservations, as outlined above.

# Response

**Published:** 2024-01-17

**Authors:** ORIANA RIVERA LOZADA

**Affiliations:** Universidad Nacional Mayor de San Marcos, Lima District, Lima Region, Peru

My main drawback with respect to the systematic analysis of the literature is in the selected databases. There is a clear sample selection bias towards Latin American and Hispanic journals as a consequence of the inclusion of the Redalyc index, since almost 50% of the bibliographic references found come from this repository.

 Do the authors think that their results could be generalized to all countries and to any time?. Would the resulting papers selected change if other repositories had been used?

**R/ The repositories used for the research were, in order: Scopus, Redalyc, La referencia, Base-Search.net, Web of Science, Scielo, DOAJ and Ebsco, considering that they met the criteria of fields Boolean expressions, temporal horizon and type of publication (Table 2). These repositories were filtered with the keywords shown in table 2a and it is precisely there where Redalyc presents the largest number of documents related to the keywords of interest for this research.**

**Perhaps another type of Boolean expressions in search offers repository participations different from those presented in this document. According to the above, we consider that there is no selection bias.**

 2) In some cases, the information on the characteristics of the sample of bibliographic references obtained does not allow relevant conclusions to be drawn. For example, Table 5 shows the Coverage years of the SJR index for the highlighted journals in the RBP. I understand that it is total coverage years in SJR, not the years used in the RBP. Is the information in this table really relevant? this information is only of the 5% of the RBP used?, are all the journals and all the papers included in SJR?

**R/ Accordingly, the years shown in Table 5 refer to the period of JRS coverage for journals and we show this to highlight 10 of the journals of the PBR, the ones with the highest score, thus informing the reader of the time of measurement of these journals by the JRS index and, therefore, their prestige.**

**There are 10 journals that contain 1 PBR article each, so they represent 12.04% of the PBR used.**

**Regarding the last question, not all articles are included in JRS, 44.57% of the PBR is found in journals that are measured with the JRS index, 42.16% of the PBR are articles from journals that have not been measured with this international index, so they are not found in figure 3 or table 5 and 13.26% are articles from web pages.  Thesis, a book and a paper.**

 3) Section "iii) Scientific production" summarizes the main findings of a selection of articles. It is not clear what criteria were used to select the articles explained in this section. How were they selected?, are they the most cited articles? are those published in the highest impact journals? or is it a purely random selection?

 It is not clear, therefore, how the factors explained in section iii) are select as the main factors behind the gender gap. Are these factor in scientific literature  supported by the quality of the journal?, or by the number of cites?, or whether this is simply a purely historical review?.

 In any case, I would like to congratulate the authors for their excellent work.

**R/ The research proposes to classify the systematic review of literature into four classifications: i) academic offer, ii) research policy, iii) scientific production and iv) research profile. Scientific production grouped the documents that focus their attention on scientific production by profession or by female researchers. In this perspective, the research presented in this section was presented due to its thematic relevance and proposed contributions, as it was selected from the documents with the highest citation.  This was added to the paper**
